# Modulations of corticospinal excitability following rapid ankle dorsiflexion in skill- and endurance-trained athletes

**DOI:** 10.1007/s00421-022-04981-9

**Published:** 2022-06-21

**Authors:** Nijia Hu, Janne Avela, Dawson J. Kidgell, Jarmo M. Piirainen, Simon Walker

**Affiliations:** 1 Neuromuscular Research Center, Faculty of Sport and Health Sciences, University of Jyväskylä, Jyväskylä, Finland; 2 Department of Physiotherapy, School of Primary and Allied Health Care, Faculty of Medicine, Nursing and Health Science, Monash University, Melbourne, Australia

**Keywords:** Physical exercise, Training adaptation, Stretch reflex, Transcranial magnetic stimulation, Corticospinal excitability

## Abstract

**Purpose:**

Long-term sports training, such as skill and endurance training, leads to specific neuroplasticity. However, it remains unclear if muscle stretch-induced proprioceptive feedback influences corticospinal facilitation/inhibition differently between skill- and endurance-trained athletes. This study investigated modulation of corticospinal excitability following rapid ankle dorsiflexion between well-trained skill and endurance athletes.

**Methods:**

Ten skill- and ten endurance-trained athletes participated in the study. Corticospinal excitability was tested by single- and paired-pulse transcranial magnetic stimulations (TMS) at three different latencies following passive rapid ankle dorsiflexion. Motor evoked potential (MEP), short-latency intracortical inhibition (SICI), intracortical facilitation (ICF), and long-latency intracortical inhibition (LICI) were recorded by surface electromyography from the soleus muscle.

**Results:**

Compared to immediately before ankle dorsiflexion (Onset), TMS induced significantly greater MEPs during the supraspinal reaction period (~ 120 ms after short-latency reflex, SLR) in the skill group only (from 1.7 ± 1.0 to 2.7 ± 1.8%M-max, *P* = 0.005) despite both conditions being passive. ICF was significantly greater over all latencies in skill than endurance athletes (*F*_(3, 45)_ = 4.64, *P* = 0.007), although no between-group differences for stimulations at specific latencies (e.g., at SLR) were observed.

**Conclusion:**

The skill group showed higher corticospinal excitability during the supraspinal reaction phase, which may indicate a “priming” of corticospinal excitability following rapid ankle dorsiflexion for a supraspinal reaction post-stretch, which appears absent in endurance-trained athletes.

## Introduction

Corticospinal plasticity, the ability of the brain to modify neuronal connections, is essential for learning, motor control, improved memory, and recovery from brain injury (Kantak et al. 2012). It can be modified by conditions such as visual, auditory, and proprioception information through adapting the neural connections (Pascual-Leone et al. 2005). Motor training is a process of acquiring information from external sources and accomplishing movement, which is intrinsically associated with neuroplasticity. Long-term sports training improves corticospinal plasticity for motor learning (Hötting and Röder 2013; Singh et al. 2016). Meanwhile, different categories of training such as endurance training and skill training seem to modify the neural system differently (Schlaffke et al. 2014).

Endurance training aims to increase the capacity of continuous motor output by repeating the same movement sequence and, therefore, increasing the efficiency of the movement (Barnes and Kilding 2015). Endurance training increases cognition and neuroplasticity in several brain regions such as the cerebellum, hippocampus, and cerebral cortex via different mechanisms of global affection, such as altered blood volume in the brain and lactate induces elevation of neural growth factors and, but does not alter specific motor map organization or synapse number (synaptogenesis), which is produced by motor learning (Thomas et al. 2012; Taubert et al. 2015). At the spinal level, the excitability of the motor neurons is known to adapt after long-term endurance training (Koceja et al. 2004). Following rapid toe movement, well-trained swimmers demonstrated higher spinal excitability than non-trained individuals (Ogawa et al. 2009), and similar results were later presented in endurance runners (Ogawa et al. 2012). In general, long-term endurance training results in enhanced spinal excitability and provides increased blood flow, and oxygen delivery through angiogenesis, to brain regions, but appears not to participate directly in the modulation of synaptic number or topology (Churchill et al. 2002; Taubert et al. 2015; Chen et al. 2019).

On the other hand, skill training is defined as the acquisition and subsequent refinement of novel movement sequences (Adkins et al. 2006). According to neuroimaging studies, when learning a new specific exercise (e.g., dancing, gymnastics) that triggers motor skill learning processes, greater neural networks are activated within the brain area of the focused task compared with simple movements (e.g., grasping and moving small objects) (Papale and Hooks 2018; Ungerleider et al. 2002). A transcranial magnetic stimulation (TMS)-based experiment showed that skill-trained athletes (dancing, gymnastics, and figure skating) have higher capacity for corticospinal plasticity of the test-relevant muscle (soleus) compared to endurance-trained athletes (cross-country skiing, orienteering), (Kumpulainen et al. 2015). During a single motor skill learning session, there is an increase in corticospinal excitability in the area controlling the corresponding limb (Suzuki et al. 2012), which may be related to decreasing cortical inhibitory neurotransmission (Kolasinski et al. 2019). From previous studies, both single session of skill training seems to modify cortical behavior, and long-term skill training has been shown to result in changes of corticospinal excitability and different cortical responsiveness versus endurance training (Suzuki et al. 2012; Perez et al. 2004).

One method to investigate the effects of interventions on corticospinal plasticity is the stretch reflex test, where neural responses are recorded by surface electromyography (EMG) (Hagbarth 1967). Such a method can be combined with stimulation methods, such as TMS, to determine the contribution of different parts of the neural system to the reflex response (Budini et al. 2017). This stretch reflex contraction occurs naturally in locomotion (i.e., stretch-shortening cycle), but the exposure to and fatigue induced by these actions during training (Avela and Komi 1998) could be hypothesized to be different between skill- and endurance athletes leading to differential corticospinal responses. For a healthy human, an imposed dorsiflexion of the ankle joint leads to a series of clear responses in the EMG of the stretched muscles. The main response, with an onset latency at 40‒50 ms, is called the short-latency stretch reflex (SLR) and is mediated by a monosynaptic reflex loop (Fellows et al. 1993; Lee and Tatton 1975). The classic view is that SLR is “purely” under spinal control, whereas only the long-latency reflex (LLR ~ 90 ms) (Dietz et al. 1984) can be influenced by cortex behavior (Petersen et al. 1998) based on latencies likely for a transcortical loop (Evarts 1973). This view is supported by TMS-evoked MEP responses not affecting SLR or medium-latency reflex (MLR), but being facilitated at LLR_2_ (after ~ 120 ms) (Taube et al. 2008). Based on the movements performed during training, e.g., endurance athletes performing repetitive stretch–shortening cycles while skill athletes perform regularly changing movement patterns, it is reasonable to assume that skill- and endurance athletes differ in their corticospinal control of movement and that this may become apparent in different phases following muscle stretch.

The aim of the current study was to explore the contribution of/and the underlying corticospinal mechanisms mediating motoneuronal responses to stretch reflex of skill- and endurance-trained athletes by recording MEPs in the soleus muscle. Both skill and endurance training are known to lead to neuronal adaptation and mechanisms of neuronal modulation in short-term sports training has been explored (Kolasinski et al. 2019), but there are contentions about how the different types of long-term training affects neuroplasticity. Whether muscle stretch influences corticospinal facilitation/inhibition differently in endurance- and skill-trained athletes and, thus the mechanism(s) behind natural movement remain unknown. It was hypothesized that endurance-trained athletes would show more prominent modulation at SLR, while skill-trained athletes would show higher modulation after SLR (SLR + 120 ms).

## Methods

### Participants and ethical approval

Ten endurance-trained athletes: seven males and three females (mean ± standard deviation: 25 ± 3 years, 70 ± 9 kg, 176 ± 8 cm) and ten skill-trained athletes: 1 male and 9 females (22 ± 3 years, 67 ± 8 kg, 165 ± 8 cm) volunteered to participate in this study. There is evidence showing that no difference exists in resting MEP between males and females (Pitcher et al. 2003). Thus, the different contribution of genders between the groups should not bias the results. Training background information was collected by a questionnaire. The endurance group had trained endurance sports on average 12 ± 3 years for 11 ± 3 h per week. Three participants practiced cross-country skiing, two long-distance running, three triathlon, and two swimming. The skill group had trained skill sports on average 13 ± 3 years for 9 ± 1 h per week. Four participants practiced aerobic gymnastics, three esthetic group gymnastics, two martial arts, and one dancing. None of the participants had any history of neuromuscular or orthopedic diseases and all participants were informed about the procedures and gave written informed consent. The study was approved by the ethics board of the university and the study was performed in conformity with the Declaration of Helsinki. Participants were asked not to train 12 h before measurements and not have any caffeine on the measurement day to avoid interference with the TMS protocol (Turco et al. 2020).

### Experimental design

There were two test sessions in this study, a single-pulse session and a paired-pulse session in that order. Before the first testing session, subjects were familiarized with both TMS and the ankle perturbations. On each test occasion, participants were positioned on a custom-built ankle dynamometer (University of Jyvaskylä, Finland) with the hip at 120° and the right knee in a fully extended position of 180°. The right foot ankle was set at 90° and rested on a pedal of the dynamometer. A seat belt restricted movement of the upper body and straps secured the right thigh and foot. Hands were resting and held together during the measurement. After the positioning procedure, the maximum compound action potential (M-max) of the resting soleus muscle was measured first. The participant contracted the ankle submaximally several times for warmup and then performed three maximal isometric plantarflexion actions with 2 min rest between trials. The highest force value from the three trials was considered as the maximal voluntary contraction (MVC). Resting stretch reflex of the soleus muscle (10 trials) and TMS (10 trials) was performed separately to calculate the latency of SLR and the latency of MEP before the experiment trials. This allowed precise arrival of the MEP to the soleus muscle coinciding with the desired stretch reflex latencies for each individual participant. During separate test sessions, MEPs of soleus muscle were elicited in four conditions: at the beginning of the pedal movement (Onset), at SLR (SLR), 120 ms after SLR in a passive condition (p120), and 120 ms after SLR while plantar flexing the ankle to 25% of MVC (a120). All single-pulse trials were performed during one test session and then following 5 days, all paired-pulse trials were performed in a second testing session. One endurance subject completed the paired-pulse but not the single-pulse testing session, meaning *n* = 10 for paired-pulse but *n* = 9 for single-pulse data in the endurance group.

### Recordings

EMG measurements were performed by bipolar electrodes (Blue Sensor N, Ag/AgCl, 28 mm^2^, Ambu A/S, Ballerup, Denmark) placed 2 cm below the gastrocnemius on the line of the Achilles tendon for soleus muscle (SOL) and over the belly for tibialis anterior muscle (TA) at 1/3 of the distance between the fibula and medial malleolus. A reference electrode was placed on the ipsilateral medial malleolus. Before electrode placement, skin under the electrodes was shaved, abraded with sandpaper, and cleaned with alcohol to reduce the resistance below 5 kΩ. EMG signals were amplified (1000 ×) by a preamplifier (NL824; Digitimer, Welwyn Garden City, UK), and then band-pass filtered (10‒1000 Hz) by another preamplifier (NL900D/NL820A; Digitimer Ltd., UK). Reaction forces from the dynamometer pedal were measured by a piezoelectric crystal transducer (Kistler Holding, Winterthur, Switzerland). EMG was sampled at 5 kHz and reaction forces were sampled at 1 kHz via a 16-bit AD converter (CED power 1401, Cambridge Electronics Design Limited, UK). Spike2 software (CED, Cambridge, UK) was used for all online data collection and offline analyses.

M-max was measured for MEP normalization purposes. M-wave was elicited with an electrical stimulator (DS7AH, Digitimer Ltd., Hertfordshire, UK) in the right soleus muscle by stimulating the posterior tibial nerve. The stimulus was a square-wave pulse of 1 ms duration. The anode electrode was placed above the patella. The cathode was placed in the popliteal fossa and moved until the best position for eliciting the M-wave with participants in standing position was found. It was then fixed to that position throughout the experiment. The M-max was tested in the experimental position and a further 20% of current was used once a plateau in response was observed (120% M-max stimulation intensity).

### TMS stimulations

TMS was delivered using a paired-pulse Magstim 200^2^ stimulator with a double cone coil (Magstim, Whitland, UK). To investigate corticospinal excitability, single-pulse TMS with 120, 140, and 150% intensity of resting motor threshold (rMT) were delivered during the four conditions. To investigate intracortical facilitation/inhibition, short-interval intracortical inhibition (SICI), intracortical facilitation (ICF), and long-interval intracortical inhibition (LICI) were measured during the four conditions. SICI was elicited by paired-pulse TMS stimulation with a suprathreshold TMS pulse (120% intensity of rMT) after a subthreshold TMS pulse (80% intensity of rMT) at 3 ms inter-stimulus interval. Similarly, ICF (15 ms inter-stimulus interval) and LICI (50 ms inter-stimulus interval) were produced using the same sub- to suprathreshold intensities (Kujirai et al. 1993; Ziemann et al. 1996; Wassermann et al. 1996).

The optimal TMS stimulus site for the right soleus muscle was located on average 1 cm lateral (left) and 1 cm posterior to the cranial apex. Several stimulations were delivered to determine optimal coil placement and it was then marked by a marker pen on the scalp of the participant. rMT was defined as the lowest stimulus intensity to elicit clear MEPs in three out of five trials. Ten TMS stimulations with 120% of rMT intensity were delivered to calculate the latency of MEP. In the single-pulse session, ten TMS stimulations were given with different intensities (120, 140 and 150% of rMT) randomly for the four conditions. There were 5–8 s intervals between each TMS stimulation in each trial and 2 min rest between conditions. In the paired-pulse session, each condition included ten TMS stimulations with 120% rMT single-pulse as the test MEP, and different paired-pulse paradigms (SICI, ICF and LICI). In passive trials, participants were asked to perform an attention task, which consisted of counting down from 200 silently. In active trials, participants were asked to focus on a line marking 25% MVC on a screen in front of them and perform plantar flexion to follow the force line throughout the trial.

### Stretch reflex induced by rapid ankle dorsiflexion

The stretch reflex of the right soleus muscle was elicited by a motor-driven ankle dynamometer (Faculty of Sport and Health Sciences, University of Jyväskylä, Finland) with dorsiflexion (rotational magnitude: 4°, speed: 3.5°rad/s). Stretch reflexes were measured while participants sat relaxed in the dynamometer chair. When ten stretch reflexes were measured, the latency of SLR was calculated in Spike2 software using the average of the waveforms. The latency of SLR was defined as the time between the onset of a digital trigger of pedal movement and the start of the ascending EMG signal.

### Data and statistical analyses

In the single-pulse session, the peak-to-peak amplitude of the soleus MEPs and stretch reflex were determined, averaged over the ten trials, and normalized to M-max. MEP amplitude from stimulations with 120, 140 and 150% of rMT, respectively, did not differ between groups. Consequently, during off-line analyses, the data from all stimulus intensities were averaged and defined as ‘MEP_AVG_’, thereby increasing the number of trials per condition to 30. MEP_AVG_ at SLR condition was compared with stretch reflex values without stimulation to demonstrate the SR/ MEP_AVG_ ratio. In the paired-pulse session, the peak-to-peak amplitude of conditioned MEP was compared to the test MEP. SICI, ICF, and LICI were expressed as a percentage of the test MEP with the following formula: (conditioned MEP/ test MEP) × 100. A higher ICF percentage represents more facilitation, while higher SICI and LICI percentage values represent less intracortical inhibition when comparing conditions.

Statistical analyses were conducted using IBM SPSS 20.0 (SPSS, Chicago, USA). All variables were processed by log transformation prior to statistical analyses, which resulted in the data being normally distributed as assessed by Shapiro–Wilk’s *W* tests. Baseline differences between the groups for training years, MVC, M-max, stretch reflex, and rMT were tested by independent sample *t* tests. TMS-induced responses were assessed by a two-way repeated measures ANOVA with within-subject factor of four levels (Onset, SLR, p120, and a120) and between-subject factor groups of two levels (endurance and skill). Mauchly’s test was used to evaluate sphericity, and where the assumption was valid *F* values were reported with sphericity-assumed degrees of freedom and df error (i.e., F _(sphericity-assumed df, df error)_). MEP_AVG_ and LICI violated the assumption of sphericity and so *F* values were reported along with their Greenhouse–Geisser adjustments (i.e., F _(Greenhouse–Geisser adjusted df, df error)_). When a significant *F* value for Condition was observed, Bonferroni post hoc tests were run for the four conditions (Onset, SLR, p120, and a120). Effect sizes for the ANOVA main effects are reported as partial eta squared (η_p_^2^), where 0.02, 0.13, 0.26 are considered small, medium and large, respectively. Correlations between MEP_AVG_ and stretch reflex, MVC and MEP_AVG_ were analyzed for non-log transformed MEP values and stretch reflex values using the Spearman’s rank correlation test. The significance level was set at *P* = 0.05 and all results were displayed as mean ± SD.

## Results

There were no differences between groups in training years (endurance group: 11 ± 3 years; skill group: 13 ± 3 years, *P* = 0.330), rMT (endurance group: 54 ± 7% stimulator output; skill group: 47 ± 8% stimulator output, *P* = 0.055) or MVC (endurance group: 297 ± 67 Nm; skill group: 227 ± 86 Nm, *P* = 0.140).

### Single-pulse MEPs

**Fig. 1:**
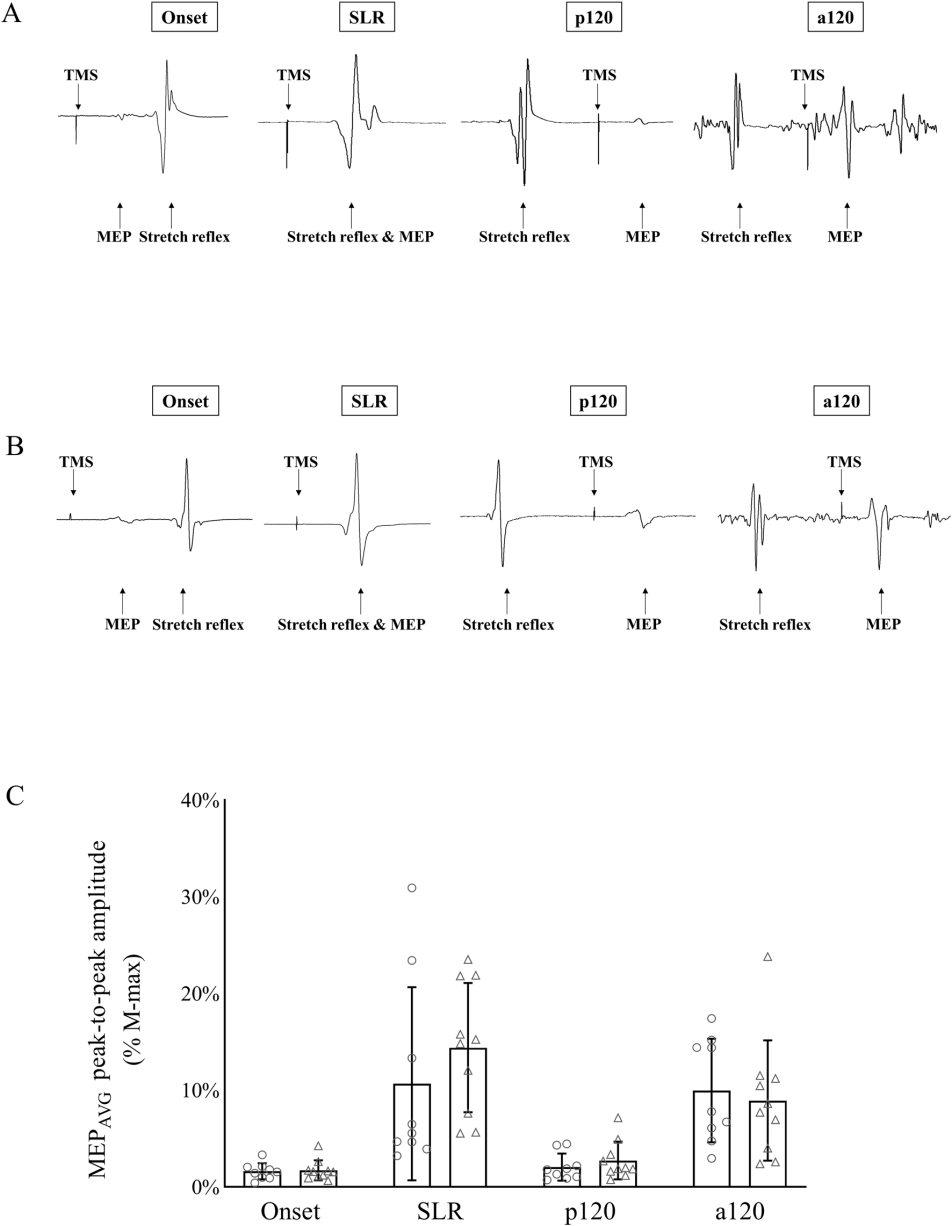
Raw EMG signals showing stretch reflex responses and MEP induced during the four conditions (Onset, SLR, p120, a120) from single TMS trials in one endurance-trained subject (**A**) and skill-trained subject (**B**). Group-level MEP_AVG_ responses during the four conditions (**C**). There was no difference shown in MEP_AVG_ between groups. Individual values are shown by symbols (open circle = endurance group, open triangle = skill group)

**Fig. 2:**
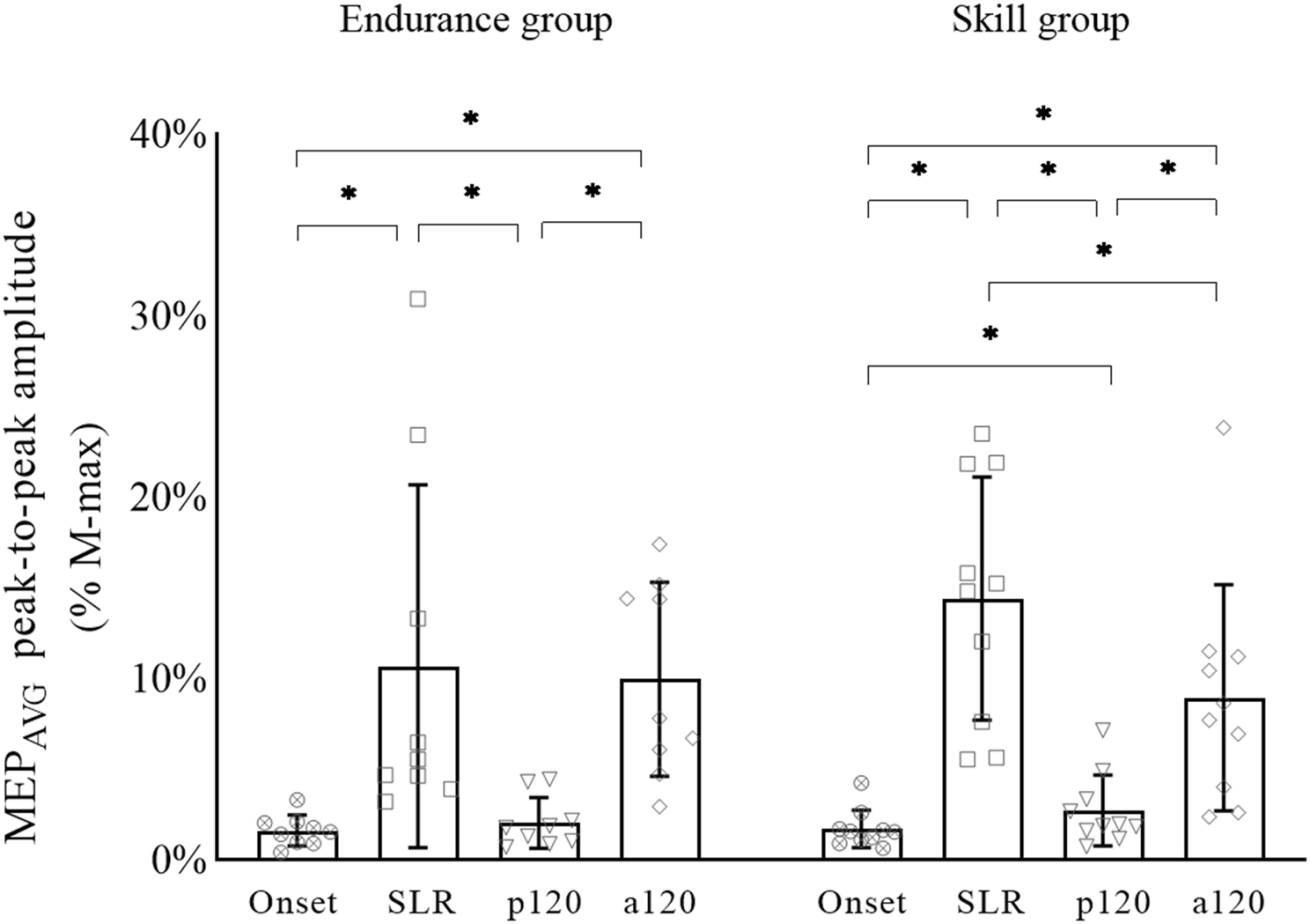
Group-level MEP_AVG_ responses during the four conditions and within-subject statistical comparisons. In the skill group, there were differences between each condition. There was no difference shown in the endurance group between Onset and p120 or SLR and a120 conditions. Values of all participants are shown by symbols for each condition (‘’ = Onset, open square = SLR, open inverted triangle = p120, open diamond = a120). asterisk = significant difference (*P* < 0.05) between conditions

A significant main effect for condition was observed (Fig. 2, *F*_(1.971, 33.51)_ = 83.908, *P* < 0.001, η_p_^2^ = 0.832), but there was no main effect for group (Fig. 1C *F*_(1, 17)_ = 0.532, *P* = 0.476, η_p_^2^ = 0.030) or group × condition interaction (*F*_(1.971, 33.51)_ = 1.88, *P* = 0.169, η_p_^2^ = 0.100). MEP_AVG_ in the endurance group was 1.6 ± 0.8%M-max (Onset), 10.7 ± 9.4%M-max (SLR), 2.1 ± 1.3%M-max (p120), and 10.0 ± 5.0%M-max (a120). MEP_AVG_ in the skill group was 1.7 ± 1.0%M-max (Onset), 14.4 ± 6.4%M-max (SLR), 2.7 ± 1.8%M-max (p120), and 8.9 ± 5.9%M-max (a120).

Significant differences over time (i.e., between conditions) were observed from Onset to SLR, SLR to p120, p120 to a120 and Onset to a120 for both groups (Fig. 2, *P* < 0.01). In addition, there was a significant difference between Onset and p120 (*P* = 0.005), and SLR and a120 (*P* = 0.024) in the skill group only (Fig. 2).

**Fig. 3:**
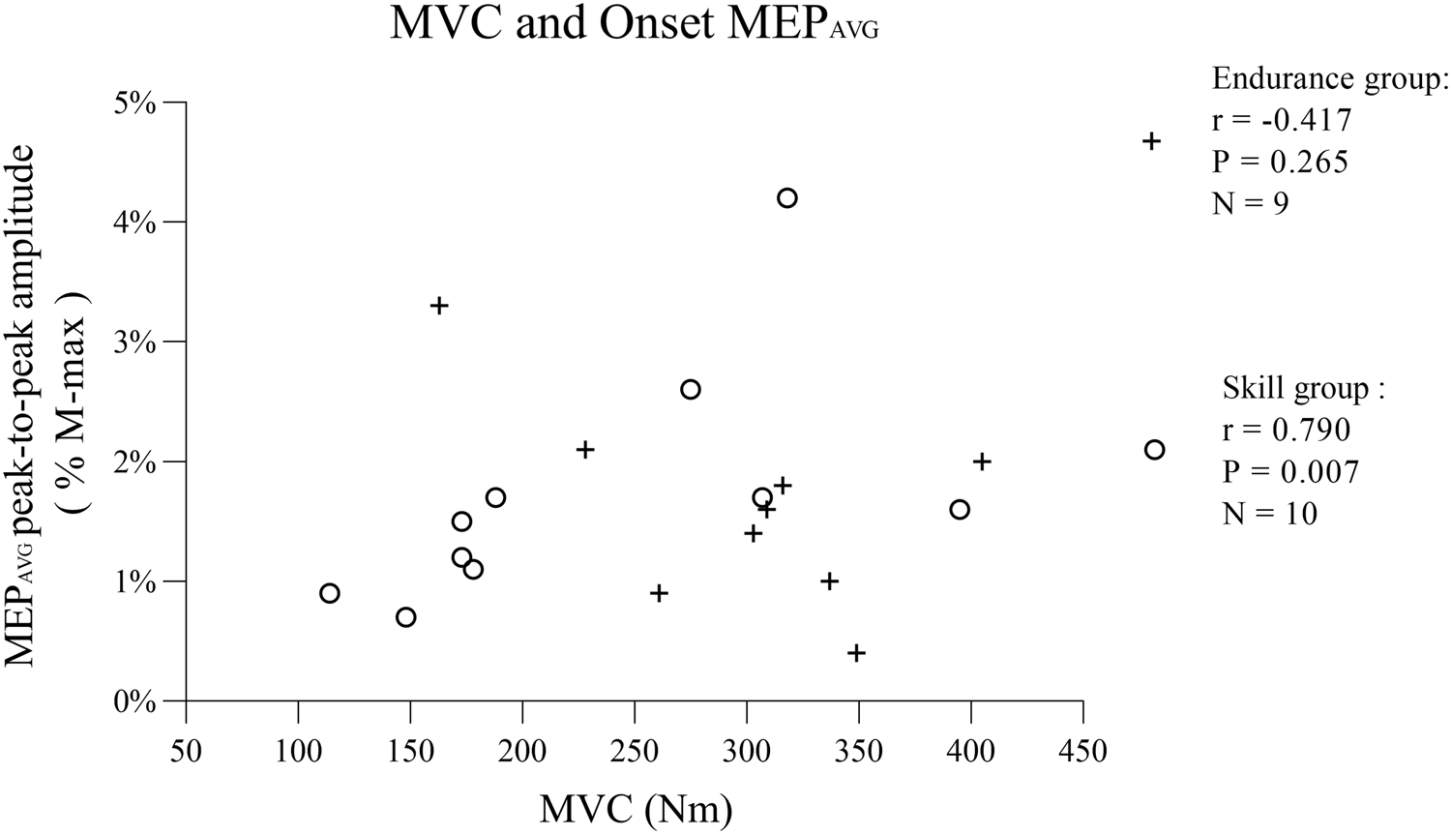
Scatter plot of MVC (Nm) and Onset MEP_AVG_ (%M-max) in two groups (endurance group = ‘ + ’, skill group = ‘○’). Data from the skill group (*N* = 10) showed a positive correlation (*P* = 0.007). Data from the endurance group (*N* = 9) did not reach statistical significance (*P* = 0.265)

There was a strong correlation between MVC and Onset MEP_AVG_ in the skill group (*r* = 0.790, *P* = 0.007, *N* = 10, Fig. 3), but no relationship was observed for the endurance group (*r* = − 0.417, *P* = 0.265, *N* = 9).

**Fig. 4:**
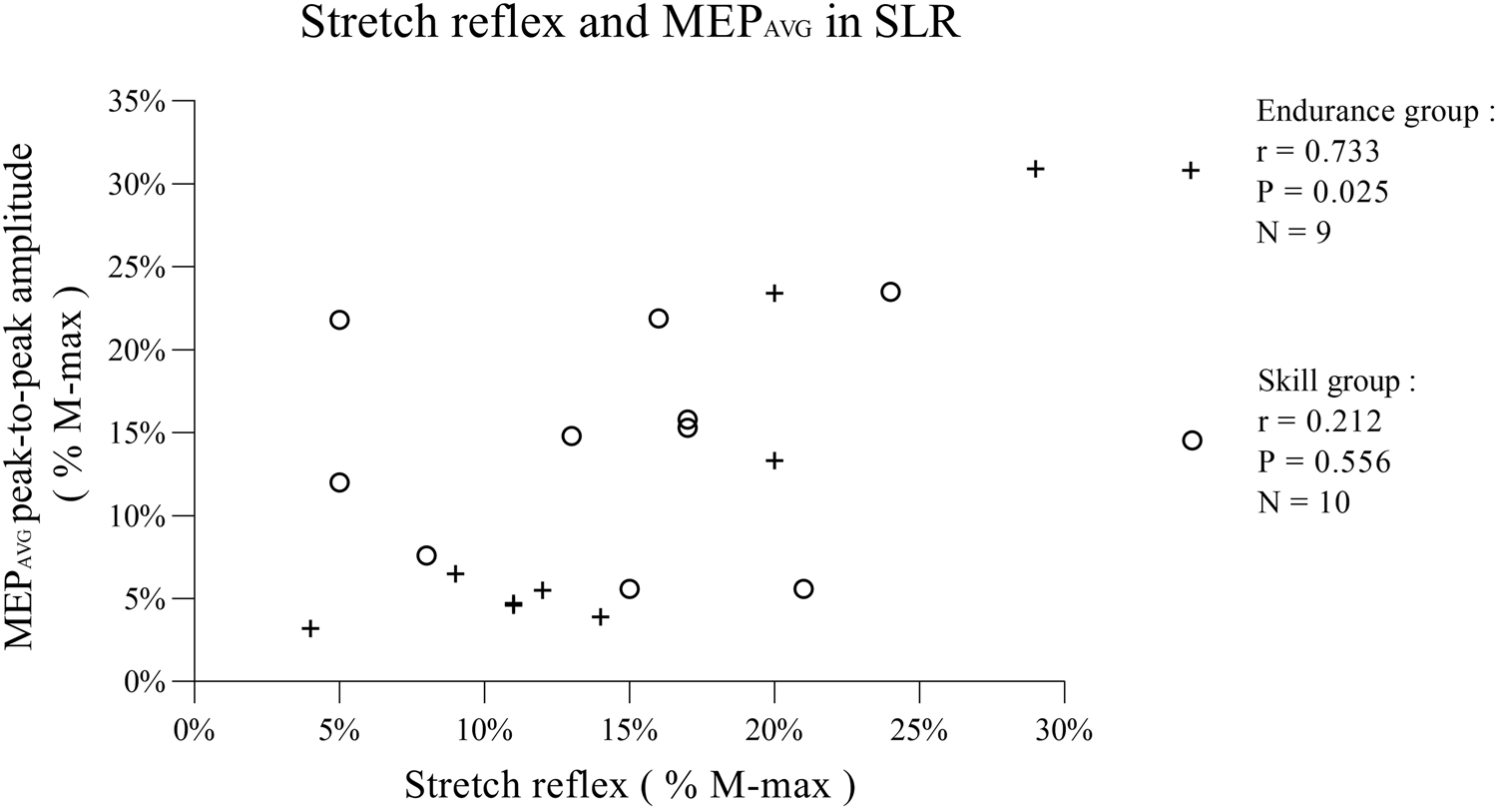
Scatter plot of stretch reflex (%M-max) and MEP_AVG_ (%M-max) in two groups (Endurance group = ‘ + ’, Skill group = ‘○’). There was a significant positive correlation observed in the endurance group (*P* = 0.025), but not in the skill group (*P* = 0.556)

SR/ MEP_AVG_ ratio revealed that the increase in MEP_AVG_ from Onset to SLR was partly affected by the presence of stretch reflex, and there were no differences between two groups (endurance = 1.8 ± 0.8; skill = 1.3 ± 1.0). However, the correlation of MEP_AVG_ and stretch reflex showed a strong relationship in the endurance group (Fig. 4, *r* = 0.733, *P* = 0.025, *N* = 9), but not in the skill group (Fig. 4, *r* = 0.212, *P* = 0.556, *N* = 10).

### Paired-pulse MEPs

SICI showed a significant main effect for condition (*F*_(3, 42)_ = 5.154, *P* = 0.004, η_p_^2^ = 0.269), but not between groups (*F*_(1, 14)_ = 0.409, *P* = 0.533, η_p_^2^ = 0.028) or group × condition interaction (*F*_(3, 42)_ = 1.074, *P* = 0.370, η_p_^2^ = 0.071). Post hoc (Bonferroni) tests for SICI did not reveal significant differences between conditions for each group separately (endurance group: Onset vs. SLR *P* = 1.000; skill group: e.g., Onset vs. SLR *P* = 0.081).

ICF showed a significant main effect for condition (*F*_(3, 45)_ = 4.64, *P* = 0.007, η_p_^2^ = 0.236) and for group (*F*_(1, 15)_ = 6.163, *P* = 0.025, η_p_^2^ = 0.291). There was no group × condition interaction (*F*_(3, 45)_ = 0.455, *P* = 0.715, η_p_^2^ = 0.029). However, post hoc tests for ICF did not show significant differences between conditions in either group.

There were no main effects observed for LICI (condition: F _(1.892, 28.386)_ = 2.186, *P* = 0.133, η_p_^2^ = 0.127; group: *F*_(1, 15)_ = 3.925, *P* = 0.066, η_p_^2^ = 0.207) (Table 1).

**Table 1:** SICI, ICF and LICI at different conditions as a percentage of the test MEP (mean ± SD)

	Paired pulse	Onset (%)	SLR (%)	p120 (%)	a120 (%)
Endurance group	SICI	50.6 ± 21.0	67.3 ± 23.0	69.4 ± 32.3	94.2 ± 35.7
	ICF	166.2 ± 90.2	97.9 ± 32.3	149.6 ± 33.8	115.2 ± 34.9
	LICI	114.3 ± 32.8	106.9 ± 29.8	141.8 ± 70.9	104.0 ± 28.2
Skill group	SICI	46.4 ± 27.9	114.8 ± 39.7	69.2 ± 37.3	86.6 ± 26.9
	ICF	185.0 ± 51.3	139.5 ± 56.4	157.7 ± 42.9	130.4 ± 35.6
	LICI	130.4 ± 45.8	128.1 ± 23.6	146.7 ± 44.8	108.9 ± 43.9

## Discussion

This study investigated changes in corticospinal excitability at different latencies relative to rapid dorsiflexion between skill- and endurance-athlete groups. As planned, the passive ankle dorsiflexion led to a stretch reflex in the soleus muscle, which is an important part of proprioceptive processing and results in afferent feedback to both spinal and supraspinal centers. It was hypothesized that the endurance-trained athletes would show more prominent corticospinal modulations at SLR, while skill-trained athletes would show higher modulation during the period where a supraspinal reaction to the movement is prominent (SLR + 120 ms). In line with the hypothesis, the present study showed higher MEPs at p120 in the skill group. However, in opposition to the hypothesis, the endurance group did not demonstrate more prominent corticospinal modulation at SLR. Finally, MVC was strongly correlated with resting MEPs in the skill group, which was not the case in the endurance group. On the other hand, a strong correlation between stretch reflex and MEPs was observed at SLR in the endurance group but not in the skill group.

In the present study, MEPs at p120 were higher than at Onset only in the skill group. p120 took place 120 ms after SLR, which was approximately at the latency of the second long-latency reflex (LLR_2_) reported by Taube et al. (2008). The increased MEPs at LLR_2_ indicated modulation of corticospinal excitability, while reduced H-reflex at the same time point suggested that this modulation was cortical in nature (Taube et al. 2008). At this phase, there is sufficient time to allow different pathways, including cortical and spinal, to contribute to the recorded MEPs’ facilitation and inhibition. Greater MEPs in the skill group 120 ms after SLR suggests that they have a greater or more long-lasting facilitation of corticospinal excitability than endurance-trained athletes after rapid ankle dorsiflexion, even in a passive condition. One important suggestion on the mechanisms of motor learning-induced cortical plasticity is that synaptic connections at the cortical level are modified through LTP (Friedman and Donoghue 2000). LTP is a prerequisite for synaptogenesis and, thus, skill training has, indeed, been shown to lead to synaptogenesis of the motor cortex (Adkins et al. 2006). PAS, which is an artificial intervention pairing electrical stimulation of somatosensory nerves and TMS of the corresponding area of the motor cortex, can produce LTP-like plasticity in the synapse. The amount of PAS-induced LTP-like plasticity increase depends on the number of active synapses. Therefore, a PAS intervention has been used as a measure of corticospinal plasticity (Lazzaro et al. 2009). In a study by Kumpulainen et al. (2015), PAS induced increased MEP in skill athletes, but not in endurance athletes or untrained adults, revealing higher corticospinal plasticity and greater synaptogenesis at the cortical level of skill-trained athletes. Previous findings suggest that skill training results in increasing adaptability of corticospinal plasticity and, thus, skill-trained athletes may preferentially rely more on cortical sources for voluntary movement as was the case in the present study at p120 and a120 condition.

It is important to note that there was no evidence of voluntary muscular activity prior to the stretch or after the stretch reflex response had abated in the passive trials (i.e., the muscle was silent). We speculate that this was a ‘priming’ mechanism in the skill group to modulate top-down responses by motor programs stored in the central nervous system after the rapid perturbation (Pierrot-Deseilligny & Burke, 2005). Possible explanations are as follows: first, for skill-trained athletes, there are more voluntary movement changes in training and competition, which need to be controlled by the motor cortex, cerebella, or somatosensory association cortex (Kurtzer 2015; Suminski et al. 2007). Second, central control has been exposited in the processing of, e.g., expected postural response (Horak et al. 1989). Thus, following the perturbation, the skill athletes may have been ‘primed’ for a voluntary response after the rapid ankle movement. In support of this contention, a strong positive correlation between MVC and resting MEPs was observed in the skill group only. MVC force is dependent on recruitment of motor units and the force-producing capacity of muscle fibers. A higher MEP value is related to higher excitability of motor cortical output cells and motor neurons during voluntary contraction (Taylor et al. 2002). Therefore, for skill-trained athletes, corticospinal excitability plays an important part in voluntary movement and is possibly observed in our enhanced p120 MEP_AVG_ during the phase where supraspinal reaction would be possible as a cortical adaptation to a top-down strategy in response to rapid ankle dorsiflexion.

Weaker, but supporting, evidence for greater reliance on cortical involvement in skill athletes was found in ICF. During all conditions, average ICF values were higher in the skill than endurance group and a significant main effect for the group was observed, indicating that ICF was higher. Nevertheless, there were no (pairwise) statistical between-group differences at any condition, which dilutes confidence in making such inferences. The cortical mechanisms of ICF are not fully clear. Increased ICF is known to be strongly influenced by decreased GABAergic inhibition or a separate increase in glutamatergic facilitation (McGinley et al. 2010; Ziemann 2003). Since the two receptors of GABAergic inhibition, GABA_A_ and GABA_B_, influence SICI and LICI, respectively (McDonnell et al. 2006; Kujirai et al. 1993), and that no differences in SICI or LICI were observed, it may be that potentially higher ICF in skill athletes was due to glutamatergic facilitation. While this is speculative, glutamatergic facilitation is one of the important molecular mechanisms for LTP, and as such, a greater ICF would support Kumpulainen et al. (2015) findings that skill athletes have higher corticospinal plasticity than endurance athletes.

Even though LICI showed facilitation (i.e. > 100% of test MEP) in the present study, this has been previously shown to occur when 50 ms inter-stimulus interval is employed in the assessment of LICI (Valls-Solé et al. 1992; Di Lazzaro et al. 2002). This presumably occurs because of increased postsynaptic excitability elicited by the conditioning stimulus or stimulus-induced activity in subcortical regions (Bolden et al. 2017; Valls-Solé et al. 1992; Di Lazzaro et al. 2002). However, the present study is not able to determine the precise mechanisms underpinning this finding.

At SLR, SR/ MEP_AVGs_ ratio was used to normalize MEPs with stretch reflex responses to reveal whether TMS has an additive effect on EMG amplitude, which was expected to demonstrate a between-group difference in the present study. However, although the ratio in each group (endurance group = 1.8, skill group: = 1.3) was raised (i.e. , > 1), no significant between-group differences were observed. In a previous study between endurance athletes and a non-trained group, endurance runners demonstrated higher monosynaptic reflex excitability by enhanced stretch reflex response, which highlights enhanced modulation of spinal excitability after long-term endurance training (Ogawa et al. 2012). In the present study, there were also no between-group differences observed for SICI or LICI at SLR. These findings imply that, in the non-motor control task (i.e., resting muscle), corticospinal modulation did not affect SLR differently between training groups.

On the other hand, SLR MEP_AVG_ showed a strong correlation with stretch reflex in the endurance group only. The monosynaptic spinal loop likely contributes more to corticospinal excitability than the supraspinal loop in the fast response phase after rapid ankle dorsiflexion. Possible reasons for the relationship in endurance athletes are as follows: training that includes repetitive stretch–shortening cycle actions was the predominant training form of eight out of this study’s ten endurance athletes (cross-country skiing, long-distance running, and triathlon). It is widely known that training-induced muscle and spinal motor neuron adaptation occurs from such a stimulus (Churchill et al. 2002; Taubert et al. 2015; Avela and Komi 1998). In rapid ankle movement, i.e., stance and swing phase in running, fast spinal loop modulation helps to keep balance during body oscillations since modulations via cortical processes would be too slow (Tahayori and Koceja 2012). In agreement with this hypothesis, a study performed using the H-reflex method revealed that after a long period of typical endurance training (well-trained swimmers in the study), athletes demonstrated greater spinal excitability (i.e., increased H-reflex) than non-trained individuals (Ogawa et al. 2009). However, the present study was unable to determine whether spinal excitability is indeed higher in endurance training athletes compared with skill training athletes through direct comparisons. There were some individuals in the endurance group seemingly specifically adapted for high excitability post-stretch from our results, and perhaps the lack of between-group differences may have been due to high within-group variance and low sample size.

Some study limitations should be considered. Due to COVID-19 and quarantine policies of the laboratory, we were only able to recruit and complete testing for 20 participants. Therefore, this sample size may not have been sufficient to determine between-group differences considering the large variabilities of MEP amplitudes when responding to rapid ankle dorsiflexion. Although it is not possible to perform a priori sample size estimation for novel measurements, the convention within the field is that typical sample sizes per group are approximately 15 (e.g., Kumpulainen et al. 2015; Wächli et al. 2017). Therefore, only the clearest differences in corticospinal plasticity between skill- and endurance athletes may have reached the level of statistical significance in the present study. This may explain, for example, significant main effects for IC, but no significant differences when post hoc tests were performed to ascertain specific differences between conditions. It is also possible that different loading patterns may induce differences in corticospinal plasticity between sports. For example, the two swimmers included in the present study may have added variance to the results given that their sport does not include stretch–shortening cycle actions in the triceps surae muscles through repetitive ground contact as in running. We suggest that sport and training characteristics should be considered when recruiting athletes as participants if the research involves corticospinal responses during motor tasks. Finally, information regarding the exact phase of the menstrual cycle was not collected in the present study. It is currently debatable whether testing during different menstrual cycle phase would influence the data (Ansdell et al. 2019; El-Sayes et al. 2019), but it may be pertinent to consider in the future.

## Conclusion

This study observed a similar pattern of corticospinal modulation, as revealed by MEP_AVG_, in long-term trained endurance- and skill athletes during and following rapid ankle dorsiflexion. However, corticospinal excitability (MEP_AVG_) was enhanced 120 ms after muscle stretch in skill-trained athletes, together suggesting a ‘priming’ of corticospinal excitability during the supraspinal reaction phase. Our skill-trained athletes demonstrated a positive relationship between MEP amplitude and MVC, supporting the view that some reliance on corticospinal excitability for voluntary action is particularly important for skill athletes.

## Data Availability

All data and materials of this study support published claims and comply with field standards.

## Abbreviations

*ANOVA*: Analysis of variance
*EMG*: Electromyography
*ICF*: Intracortical facilitation
*LICI*: Long-latency intracortical inhibition
*LLR*: Long-latency reflex
*LTP*: Long-term potentiation
*MEP*: Motor evoked potential
*MLR*: Medium-latency reflex
*M-max*: Maximum compound action potential
*PAS*: Paired associative stimulation
*rMT*: Resting motor threshold
*SICI*: Short-latency intracortical inhibition
*SLR*: Short-latency stretch reflex
*SOL*: Soleus muscle
*TA*: Tibialis anterior muscle
*TMS*: Transcranial magnetic stimulation
